# Formed on Ice: A Qualitative Study of Motivation, Pressure, and Identity in Early Ice Hockey Specialization

**DOI:** 10.3390/sports14060235

**Published:** 2026-06-08

**Authors:** Sofia Ryman Augustsson, Linnéa Kristedal Asp, Pauline Schmidt

**Affiliations:** Department of Sport Science, Linnaeus University, 391 82 Kalmar, Sweden; linneakasp@outlook.com (L.K.A.); pauline.schmidt01ps@gmail.com (P.S.)

**Keywords:** parental support, performance, social network, talent development, burnout

## Abstract

While much of the current research on early specialization focuses on physical outcomes, training models, and policy implications, little is known about how athletes themselves make sense of their developmental experiences. This study aims to examine how ice hockey players perceive and experience early specialization within competitive youth sport contexts, with the goal of generating a nuanced, inductively grounded understanding of athlete development from the athlete perspective. A qualitative study design was used where eight current and former ice hockey players with experience of early specialization participated. Data were collected using semi-structured interviews and analyzed using qualitative conventional content analysis. Three overarching themes emerged, highlighting experiences of loneliness, pressure, and elevated expectations within elite sport environments, alongside the vital importance of support networks and team community: ‘Thrown into adulthood with premature expectations’, ‘Balancing Support and Pressure in Athlete Development’, and ‘The Struggle Between Dream and Reality’. Players described feeling pressured, isolated, and prematurely professionalized, often at the expense of personal development. The findings highlight the psychological and structural challenges of early specialization in elite ice hockey. While support systems played a crucial role, they also contributed to performance anxiety and external expectations. These insights underscore the need for youth sport systems that prioritize long-term athlete well-being over short-term success.

## 1. Introduction

Ice hockey is a team sport that places high demands not only on physical performance [1] but also on psychological and interpersonal qualities such as leadership, discipline, and cooperation [2]. Currently, over 79,000 individuals are licensed within Swedish ice hockey, with the vast majority being boys and men [3]. This reflects the sport’s broad appeal and underscores the pressure faced by young players in highly competitive settings. Ice hockey also holds a prominent position in Sweden and is often regarded as one of the country’s leading national sports [4], which may further intensify expectations placed on young athletes striving for success. This cultural significance, combined with competitive selection structures, can contribute to heightened pressure to perform and achieve results from an early age.

In Sweden, the development of elite players is commonly formalized through specialized sport programs at upper secondary schools, which combine education with intensive athletic training [5]. These environments are designed to facilitate long-term athlete development and progression towards elite performance. Within this developmental context, the concept of early sport specialization, typically defined as intensive, year-round training in a single sport at a young age, often at the exclusion of other activities [6], has become a central topic of debate. While early specialization is frequently justified by its perceived advantages for talent identification and competitive success [7], a growing body of research highlights associated risks, including overuse injuries, psychological stress, and burnout [6,8,9]. This debate is often framed within established theoretical models of athlete development, particularly the Long-Term Athlete Development (LTAD) model and the Developmental Model of Sport Participation (DMSP) [10,11]. Both models advocate early diversification rather than specialization, emphasizing the importance of varied sport participation during childhood to support motor skill development, intrinsic motivation, and long-term engagement in sport. According to these frameworks, specialization should occur later in adolescence, following a sampling phase characterized by broad participation and deliberate play [11].

In addition, empirical research provides support for these models, suggesting that many elite athletes have followed diversified developmental pathways and did not specialize early [12,13]. However, despite alignment between theory and evidence, early specialization remains prevalent in practice. This discrepancy has been attributed to structural and social pressures, including parental expectations, institutionalized talent development systems, and perceived pathways to professional careers [14,15]. In organized sport contexts such as elite hockey high schools, these pressures may be further reinforced through selection processes and performance-oriented training environments.

Although previous research has primarily focused on physiological outcomes, training structures, and policy perspectives [16,17,18,19], less attention has been paid to how athletes themselves perceive and experience early specialization. Despite the prominence of LTAD and DMSP in policy and research, limited attention has been given to how these developmental pathways are experienced by athletes within structured elite environments. From a theoretical standpoint, this represents a critical gap, as both LTAD and DMSP emphasize athlete-centered development and the importance of psychosocial factors in long-term success. Understanding athletes’ subjective experiences is therefore essential for evaluating the practical implications of these models and for informing development systems. Accordingly, this study aims to examine how ice hockey players perceive and experience early specialization within competitive youth sport contexts, with the goal of generating a nuanced, inductively grounded understanding of athlete development from the athlete perspective.

## 2. Materials and Methods

### 2.1. Study Design and Participants

This study used a qualitative interview design with an inductive approach to explore ice hockey players’ experiences of early specialization. Participants were current or former male ice hockey players aged 18–30 who met two inclusion criteria: (1) early specialization and (2) experience of competing at an elite level in Swedish league play, either currently or in the past. Elite level was defined as senior competition in HockeyAllsvenskan (HA) or the Swedish Hockey League (SHL), or junior-level participation in J18 or J20 Elite [20]. A purposive sampling strategy was applied, using criterion sampling to recruit information-rich cases for in-depth exploration of experiences [21,22]. Early specialization was defined based on two of Côté’s [23] three stages of Developmental Model of Sport Participation, specifically the specializing years (ages 13–15), involving a narrowing to one or two sports, and the investment years (from around age 15), reflecting full commitment to elite performance. Exclusion criteria included players who had not specialized before 15 or lacked elite-level experience in Sweden. Recruitment began by emailing eight elite hockey clubs, targeting sports managers and communication staff. Two clubs declined participation, after which social media was used to broaden recruitment, and a HA representative shared the information letter with active players. A total of eight participants were ultimately recruited and included in the study. No participants withdrew after agreeing to participate. The relatively small sample size reflects the challenges associated with recruiting elite athletes with experience of early specialization, as access to this population is limited and participation is dependent on availability and willingness.

### 2.2. Procedure

Interviews were conducted between February to March 2025, either in person (n = 2) or via video link (n = 6) using Zoom (Zoom Communications, Inc., San Jose, CA, USA) or Microsoft Teams (Microsoft Corporation, Redmond, WA, USA). All sessions were audio-recorded and supplemented with field notes to support transcription and analysis. Data were collected through semi-structured interviews guided by a self-developed interview guide. Each interview lasted 30–60 min and addressed topics including players background, hockey career trajectory, and perceptions of early specialization.

### 2.3. The Interview Guide

The authors developed a semi-structured interview guide comprising questions across four main topics: (1) The early hockey career; (2) experiences and perceptions of early specialization; (3) social support and motivation; and (4) reflections and the future (Supplementary file). The interview guide was developed based on prior research and informed by expert consultation. The research team consisted of sports scientists with expertise in talent development, high-performance coaching, and the role of the coach within performance environments. In addition, one author (SRA), a sports physical therapist, contributed practical and clinical expertise related to youth athlete development, burnout, and injury. A pilot interview was conducted with one subject to test the guide and identify any potential shortcomings. Minor adjustments were made to the guide based on feedback from this pilot interview.

### 2.4. Data Analysis

Data were analyzed using conventional qualitative content analysis with an inductive approach [24]. This approach was selected to capture players’ experiences and meanings grounded in their own narratives, without imposing pre-existing frameworks.

Categories were not intended to verify existing theories. Instead, themes were expected to emerge naturally from the data. Following transcription, all interviews were read several times to achieve immersion and a sense of the whole [22]. The analysis was conducted iteratively, involving repeated movement between the data, codes, and emerging categories to refine interpretations and ensure that findings remained grounded in the data. Initial impressions and reflections were noted throughout this process. Meaning units relevant to the study aim were identified across the transcripts, including both recurring and divergent experiences. These meaning units were condensed while preserving their core meaning and subsequently labelled with codes derived directly from the data. The codes were continuously compared for similarities and differences and grouped into categories representing the manifest content. Through further abstraction and iterative interpretation, overarching themes were developed to capture the latent content, reflecting underlying meanings beyond the explicit statements [24].

To enhance trustworthiness, all authors were involved throughout the analytic process, and the study followed established qualitative criteria, including credibility, dependability, and confirmability, as outlined by Lincoln and Guba [25] and further applied in qualitative content analysis [26]. Regular discussions were held to critically review codes, categories, and themes, and to challenge alternative interpretations. Credibility was approached by prolonged engagement with the data, involving repeated readings and an iterative analytic process to ensure that interpretations remained grounded in participants’ accounts. Both similarities and variations in the data were actively sought and included in the analysis. Representative quotations are presented to illustrate the connection between the data and the findings. Dependability was addressed through transparent reporting of the research process, including data collection procedures, coding, and category development. Confirmability was strengthened through researcher triangulation, whereby all authors were involved in the analytic process and engaged in ongoing discussion and critical reflection. Codes, categories, and themes were reviewed collaboratively, and interpretations were continuously questioned to reduce the influence of individual bias. The analysis proceeded until consensus was reached and a coherent structure that adequately represented the data was established [27]. Together, these strategies aimed to ensure that the findings accurately represent the participants’ experiences and are grounded in the data rather than shaped by preconceptions. Examples of the analytic process are presented in Table 1.

## 3. Results

Eight ice hockey players participated in the study, representing HA (n = 5), Hockeyettan (n = 1), and Division 3 (n = 1), with one no longer active in league play (n = 1). They had all participated in elite level in Swedish league play or at elite junior-level. Based on their experiences and perceptions of early specialization, three overarching themes and ten categories were identified: ‘*Thrown into adulthood with premature expectations*’, ‘*Balancing Support and Pressure in Athlete Development*’ and ‘*The Struggle Between Dream and Reality*’ (Table 2). To maintain confidentiality, pseudonyms are used to replace players’ real names.

### 3.1. Thrown into Adulthood with Premature Expectations

The following theme describes the feelings and experiences associated with the identity of ‘hockey player’ and was shaped by four categories; *Forced Maturity*, *Hockey bubble and identity*, *Loneliness within the team* and *Structural demands and cultural narratives.* This theme captures the experience of young players, particularly around the age of 15, being abruptly thrust into adult roles and expectations within elite sport settings. It reflects how participation at a high level often requires adolescents to navigate mature environments, such as locker rooms and competitive hierarchies, long before they are developmentally prepared.

These quotes capture the unintended trade-offs and psychological impacts of early and intense involvement in elite sports (Table 3). They reflect a lack of informed choice at a young age, where players commit to a path before they are developmentally ready to evaluate their long-term interests. Adam highlights how early choices and commitments in sports can lead to other opportunities being unconsciously passed up at a young age. The sense of being “in a bubble” or “just going with it” suggests a loss of agency and awareness, while reflections on being “spoiled” highlight the difficulty of adjusting to life outside the structured, performance-driven world of elite athletics. Collectively, these voices underscore the long-term consequences of early specialization, not only in missed opportunities, but also in the challenges of transitioning to everyday life beyond sport.

Players also reported experiencing constant pressure that extended beyond physical training, encompassing the psychological strain of continuous performance. It was also noted that loneliness became more pronounced when the team community, often a fundamental aspect of a player’s identity, dissolved, resulting in a more isolated individual experience. In such contexts, the team environment remained the only perceived source of safety and belonging.

Gustav and Lukas reflect on the demands and expectations of elite sports, which they see as conditions to accept. Axel and Joel highlight the importance of long-term athletic development rather than early peak performance. Their view challenges the focus on early success in ice hockey, advocating a more sustainable, gradual approach to growth. The quotes also show that systemic structures and cultural expectations drive early specialization, often beyond players’ preferences or readiness. Together, these perspectives reveal a critical awareness that the elite sport system prioritizes short-term success over long-term development and well-being. Players recognize that, although early specialization is not ideal, structural and cultural pressures make it seem inevitable.

### 3.2. Balancing Support and Pressure in Athlete Development

Having a supportive network is frequently identified as a key aspect of player development. However, support is not always experienced positively; in some cases, it was perceived as pressure that impedes rather than facilitates progress. This theme includes the categories: *Parents’ dream*, *Extrinsic motivation and pressure*, and *The hockey dad*. The theme underscores the dual role of parents as both a source of external pressure and a provider of support. It also highlights the players’ perception that they were living out their parents’ dreams (Table 4).

The findings also emphasize that hockey frequently becomes the primary focus, and many parents may lack the requisite understanding to offer effective support. Additionally, this dynamic was reflected in partner relationships, which were reported to be strained, particularly when one party was less involved than the other. A common, often unconscious expectation is that a father should feel pride in his child’s achievements. This is communicated through subtle praise, encouragement, or comments implying that success validates the father. This dynamic can create pressure to perform, not just for the player’s growth but to meet parental expectations. Consequently, achievements may be tied more to the father’s pride than the player’s own goals. However, support feels strongest when it comes from genuine encouragement, like a father who values enjoyment throughout the athletic journey.

### 3.3. The Struggle Between Dream and Reality

As a young ice hockey player, harbouring dreams of a career in the National Hockey League (NHL) is a common occurrence. These dreams remain, but over time the distance between the dream and reality becomes increasingly palpable. The following theme illuminates the thoughts and feelings that arise in connection with these future dreams, as well as the critical moment when the dream no longer seems attainable. It also highlights the importance of long-term athletic development in contrast to early sport specialization, alongside an acceptance of the structural expectations inherent in elite sport. It emerged from three categories: *The NHL dream*, *Reality check*, *Self-Doubt and Performance Anxiety* (Table 5).

Axel and Lucas express the insight that an athletic career is both short and uncertain. Lucas reflects on the limited timeframe and together, their statements illustrate the difficult trade-offs young players face. The players emphasize their personal strategies for coping with adversity, while also reflecting on how former teammates have navigated similar challenges. Together, their accounts illustrate how the drive to succeed may be both reinforced and tested as competitive pressures increase. For most, indications of unconscious performance anxiety were apparent, whereas a smaller number explicitly articulated a clear sense of pressure.

## 4. Discussion

This study employed a qualitative, inductive methodology to investigate ice hockey players’ experiences and perceptions of early specialization. Analysis of the interview data revealed three primary themes, which underscored feelings of loneliness, pressure, and heightened expectations within elite sport environments, as well as the critical role of support networks and team community.

### 4.1. Thrown into Adulthood with Premature Expectations

This theme highlights the tension faced by youth players who enter elite sport pathways before they have the cognitive, emotional, or social maturity to make informed life choices. Their experiences show how early specialization can limit identity exploration and close off other life options. As Adam notes, fourteen-year-olds are still forming their identities and often unaware of long-term consequences. The “bubble” metaphor suggests a narrow world where players go along rather than make reflective decisions, echoing Arnett’s concept of emerging adulthood [28], where delayed exploration may lead to later disorientation or unpreparedness. Pelle’s comment about struggling with the idea of a “regular job” further illustrates how the highly structured and reinforced environment of elite sport may inadvertently inhibit the development of transferable life skills or realistic self-concept outside of sport. These reflections suggest that early sport specialization may hinder adaptive identity development, leaving individuals vulnerable to identity disruption when their athletic role is challenged or ends. Recent research has further emphasized that early specialization may impact not only physical development but also psychological well-being and identity formation, particularly when athletes are required to commit to performance pathways at an early age [29].

The statement “*It’s just constant pressure, it never lets up*”, conveys an internal monologue marked by stress and fatigue, establishing a central theme of persistent pressure within the context of elite sports. This aligns with recent research highlighting the psychological demands associated with progression in elite sport systems, where increasing expectations and competitive pressure may negatively impact athlete mental health when not adequately supported [30]. This pressure extends beyond the physical demands of training to encompass the psychological burden of continuous performance, self-improvement, and the need to meet both personal aspirations and external expectations. The findings reveal a critical tension between players’ personal perspectives on development and the structural realities of elite sport systems. While several players, such as Gustav and Joel, expressed skepticisms toward the necessity of early specialization, their narratives also acknowledged that the existing system implicitly demands it. Gustav’s remark that “the system is somewhat built for you to specialize” reflects the way institutional structures and long-standing cultural narratives reinforce early commitment, even when players themselves question its value.

From a structural functionalist view, elite sport functions as a system built to efficiently produce performance outcomes by sorting players early. This logic often emphasizes short-term success, creating high-performance environments where individual readiness is secondary to results. Lucas’ comment, “In professional sports, it just doesn’t work like that”, highlights the shift from inclusive, recreational sport to a meritocratic model demanding quick adaptation and early excellence. Axel’s critique of the “Elite Focus” program suggests that early labelling can create premature pressure. Joel’s view that “you should be peaking at 30, not at 16”, supports LTAD models [11,18] emphasizing delayed specialization and development aligned with biological and psychological growth. However, the present findings also highlight a discrepancy between these theoretical recommendations and the practical realities of elite sport systems, where early specialization appears structurally embedded.

In sum, these insights show how players navigate a system where cultural norms, structural pathways, and unspoken expectations make early specialization seem not just common, but necessary. This normalization can mask risks like burnout, identity foreclosure, and limited social growth, even as some players remain critically aware. The gap between what players see as ideal and what the system demands raises questions about how talent development models are applied, and whether they truly support holistic, long-term athlete development.

### 4.2. Balancing Support and Pressure in Athlete Development

This theme highlights the disconnect between public perception and the internal challenges these youths face, including emotional, social, and identity-related pressures that may go unnoticed by those outside the elite sport context. The pressure can become overwhelming even at an early age, particularly when it originates from multiple sources, including individuals who lack a meaningful connection to the player. The findings from this theme illuminate the complex and often contradictory role of parental support in the development of young ice hockey players. While a strong support system is commonly seen as a facilitator of athlete success [31,32,33], the results suggest that support, particularly from parents, can simultaneously serve as a source of pressure. This duality aligns with previous research on parental pressure, indicating that parental involvement, though well-intentioned, may contribute to heightened stress and diminished intrinsic motivation when it becomes overbearing or misaligned with the athlete’s personal goals [34]. However, the present findings further indicate that athletes themselves actively interpret and negotiate these expectations, suggesting a more complex and dynamic role of parental influence than previously described.

Players’ narratives often reflected a sense of fulfilling parental dreams rather than their own aspirations, particularly in relation to their fathers. The motif of the “hockey dad” emerged as a significant figure, both as a source of encouragement and as a symbol of performance-related expectations. This mirrors research on sport parenting which emphasizes that when athletes perceive parental pride as conditional on performance, it can lead to internalized pressure and self-worth tied to achievement [35]. This finding extends previous work by suggesting that athletes actively interpret and negotiate parental expectations, highlighting a more dynamic and relational process of influence than is often described in the literature.

The statements reveal that some parents lack understanding of elite sport’s demands, which can increase players’ psychological strain, especially when expectations are unrealistic, or communication is weak. These tensions also affect romantic relationships, strained by the time, energy, and commitment the sport requires. These findings align with Wylleman and Lavallee’s [36] model of holistic athlete development, which emphasizes the importance of both athletic and psychosocial factors in athlete well-being. Despite these pressures, the data suggest that parental support rooted in genuine care and a focus on enjoyment can help protect against burnout and performance anxiety. This supports self-determination theory [37], which holds that autonomy-supportive environments, those fostering intrinsic motivation and emotional safety, promote lasting engagement and satisfaction. Overall, the findings point to the need for more nuanced parent education in elite youth sport. Encouraging emotionally supportive, autonomy-focused, and realistic parental behaviors may not only boost performance but also support long-term mental health.

### 4.3. The Struggle Between Dream and Reality

The lived experiences of young ice hockey players captured in this theme speak to a profound and often painful journey between aspiration and acceptance. At its core, the narrative traces how dreams of reaching the NHL, a symbol of ultimate success, are gradually tempered by the realities of elite sport, exposing a stark tension between youthful idealism and the demands of structured, high-performance environments.

For many, the dream of playing in the NHL functions as both a motivational force and a central component of identity [38]. Joel’s statement, “My love for hockey will probably never fade,” represents a passion for the sport that persists even as expectations shift. However, Axel’s reflection, “you start to realize, that dream isn’t all that realistic”, highlights the common process of dream recalibration that occurs as players mature and face increasingly competitive environments. This is further reflected in Lucas’ comment: “The career doesn’t last forever, I get that, trust me,” suggesting an early understanding of sport’s limited shelf life. Such realizations align with research showing that young athletes must frequently balance idealistic goals with realistic evaluations of their chances in elite sport [39]. The emotional burden of elite sport surfaces strongly in players’ narratives, often in the form of performance anxiety, fear of failure, and self-doubt. Ville’s observation, “you’re not the star anymore,” illustrates how identity disruption can occur when a previously high-performing athlete encounters a more competitive peer group [40]. These transitional phases are often poorly supported and can negatively affect self-esteem and intrinsic motivation, especially in environments that emphasize results over development [30,41]. In addition, this reflects findings from recent research showing that identity disruption is a common experience among athletes progressing through elite systems, particularly when performance expectations shift or competitive demands increase [30,42,43].

While previous research has primarily focused on dropout as an outcome, the present findings provide insight into how these processes are experienced and interpreted by athletes themselves. Adam and Gustav reflect on peer dropouts and how they stayed motivated, highlighting systemic attrition in youth sport. Research shows many athletes quit not from lack of talent, but due to burnout, low enjoyment, or feeling inadequate [44]. More recent research on youth sport dropout similarly highlights how psychological pressure, reduced enjoyment, and perceived lack of competence contribute to disengagement from sport [45]. Early specialization can intensify these issues by limiting identity growth and increasing stress. As the players note, those struggling with injuries, development, or mental health often feel forced to leave the sport. Viewed together, the players’ narratives reveal a complex emotional landscape in which passion, pressure, and pragmatism intersect. Their experiences highlight a persistent tension between long-term development and the structural demands of elite sport systems, where pathways to success are both narrow and demanding. These insights raise important questions about how young athletes are supported, particularly in relation to identity development and psychological well-being, and point to the need for environments that promote more sustainable and holistic development. Recent research continues to emphasize the importance of integrating psychological and developmental perspectives into talent development systems, further reinforcing the relevance of the present findings.

Taken together, the findings of this study provide a nuanced, athlete-centered perspective on early specialization within competitive youth ice hockey, highlighting the complex interplay between structural demands, interpersonal relationships, and individual development. While previous research has often emphasized outcomes such as performance, injury, or dropout, the present study contributes by illuminating how these processes are experienced and interpreted by athletes themselves. The results underscore a tension between theoretical models advocating gradual development and the realities of elite sport systems, where early specialization is often normalized and structurally reinforced. Moreover, the findings point to the importance of supportive environments that balance ambition with psychological well-being, allowing for identity development beyond athletic performance. In this sense, the study extends existing literature by demonstrating that early specialization is not merely a training pathway, but a lived experience shaped by expectations, relationships, and evolving self-understandings, with important implications for how talent development systems are designed and implemented.

### 4.4. Limitations

A limitation of this study is the relatively small sample size, which may restrict the range of experiences captured and limit the transferability of the findings to other contexts. However, consistent with qualitative research, the aim was to achieve depth of understanding rather than breadth. The study draws on internal generalization, providing rich and context-specific insights rather than aiming for statistical generalization [46]. Trustworthiness was addressed through considerations of credibility, dependability, and transferability [22]. It should also be noted that participants reflected on past experiences, which introduces a retrospective dimension that may influence recall. This retrospective perspective may affect the precision of specific details, as their recollections may have been influenced by subsequent impressions or the perspectives of others. Simultaneously, the passage of time may have facilitated deeper reflection, enabling a more nuanced understanding of their experiences and supporting greater meaning-making.

In addition, variations in interview conditions, including the use of video interviews, may have influenced the depth of interaction and interpretation of non-verbal cues. These factors should be considered when interpreting the findings. However, to achieve broader geographical coverage and player diversity, the authors opted to conduct some interviews via video link.

## 5. Future Research Directions and Implications for Practice

Future research should continue to explore early specialization from an athlete-centered perspective, with particular attention to how psychological, social, and structural factors interact over time. Longitudinal studies are needed to better understand how early specialization influences identity development, mental health, and career transitions across different stages of the athlete pathway. Expanding research across different sports and cultural contexts would further strengthen understanding of how these dynamics vary between environments. In addition, incorporating the perspectives of coaches, parents, and sport organizations could provide a more comprehensive view of how expectations and support systems are constructed and maintained. From a practical and policy perspective, the findings highlight the need for talent development systems that balance performance ambitions with long-term well-being. This includes promoting autonomy-supportive environments, strengthening parent and coach education, and encouraging more flexible and holistic development pathways. Such approaches may help reduce psychological pressure while supporting sustainable athlete development over time.

## 6. Conclusions

The findings highlight the complex interplay between early specialization, psychological pressure, and the sociocultural structures that shape elite ice hockey in Sweden. Players’ narratives reveal how premature professionalization can lead to identity foreclosure, emotional distress, and a diminished sense of personal agency. Although support networks, particularly familial ones, can aid development, they may also reinforce performance pressures and external expectations. Early aspirations of professional success often collide with the realities of elite sport, exposing patterns of self-doubt, performance anxiety, and the emotional cost of falling short within a rigid, high-stakes system. This persistent tension between aspirational goals, such as reaching the NHL, and the lived experience of elite sport underscores the need for more sustainable models of athlete development. The findings emphasize the importance of balancing ambition with long-term well-being in the design of youth sport systems. They also point to the need for education and clear guidelines for parents, coaches, and sports institutions to help mitigate pressure and promote emotionally sustainable athletic engagement. Ultimately, researchers and policymakers are urged to reconsider existing talent development frameworks, advocating for later specialization and more holistic support structures.

## Figures and Tables

**Table 1:** Examples of meaning units, condensed meaning units, codes, categories and themes from the present data.

Condenced Meaning Unit	Code	Category	Theme
“One day off would’ve been nice”	Burnout	Forced Maturity	Thrown into adulthood with premature expectations
“You could be at the ice rink all day”	Youthful stamina	Hockey bubble and identity	
“You’re only around people who do the same thing”	Closed social environment	Loneliness within the team	
“Thought it was normal, you didn’t really know anything else”	Normalized experience	Structural demands and cultural narratives	
“Parents push their kids too hard”	Parents pressure	Parents’ dream	Balancing Support and Pressure in Athlete Development
“My family supported me”	Family support	Extrinsic motivation and pressure	
“When I mentioned floorball, my dad got pretty upset”	Disappointed dad	The hockey dad	
“Life is too short not to follow your dreams”	Dreams	The NHL dream	The Struggle Between Dream and Reality
“I’ll never play higher”	Realization	Reality check	
“You didn´t want to get up”	Low internal motivation	Self-Doubt and Performance Anxiety	

**Table 2:** Themes and categories from the analysis.

Themes	Categories
	Forced Maturity
Thrown into adulthood with premature expectations	Hockey bubble and identity
	Loneliness within the team Structural demands and cultural narratives
	Parents’ dream
Balancing Support and Pressure in Athlete Development	Extrinsic motivation and pressure
	The hockey dad
	The NHL dream
The Struggle Between Dream and Reality	Reality check
	Self-Doubt and Performance Anxiety

**Table 3:** Exemplary quotes for the theme Thrown into adulthood with premature expectations.

Quotes (Meaning Units)	Category
“Hanging out with adult men when you were 15”—Hugo	Forced Maturity
“A little more than one could dream of, far too young and then you have to sign a contract”—Hugo	
“When you’re fourteen, you really don’t know yet what you’re going to be good at. Whether you realize it or not, you end up passing on opportunities without even knowing it”—Adam	
“I don’t know if I can manage to have a regular job, you get a bit spoiled that way”—Pelle	Hockey bubble and identity
“You’re living the dream, and then suddenly you’re in this bubble—you just go with it”—Adam	
“Honestly, it’s true, I didn’t really do much else in high school besides training”—Anton	
“I’ve always felt pressure, but it’s mostly from myself, I’ve always been my own toughest critic”—Ville	
“It’s just constant pressure—it never lets up”—Hugo	
“As soon as you’re not with the team, it feels like there’s no one you can really turn to”—Axel	Loneliness within the team
“It’s kind of like you’re always competing against your best friends—it really comes down to who wants it the most”—Ville	
“I wouldn’t say that early specialization is necessary to reach elite level; however, I do believe that the system is somewhat built for you to specialize”—Gustav	Structural demands and cultural narratives
“It’s not like youth sports where everyone gets a juice and a bun and can take part. In professional sports, it just doesn’t work like that, no matter what sport it is”—Lucas	
“They call it Elite Focus, but that doesn’t mean you’ve got to be the best already”—Axel	
“If you want a long career, you should be peaking at 30, not at 16”—Joel	

**Table 4:** Exemplary quotes for the theme Balancing Support and Pressure in Athlete Development.

Quotes (Meaning Units)	Category
“The parents want it more than the player does”—Pelle	Parents’ dream
“Parents shouldn’t be trying to live their own dreams through their kids”—Gustav	
“The fact that such great pressure is placed early can easily translate to the idea that you must	Extrinsic motivation and pressure
be the best now, and there is nothing else. You must be the best now, and that is the most important thing ahead of you”—Axel	
“In my opinion, there are frighteningly many parents who have absolutely no clue about ice hockey and what it means to be an ice hockey player”—Axel	
“What can you say, there was support but not all the way. I don’t think she was prepared for reality”—Joel	
“At one point, I called my mom and almost cried because I wanted to quit, but she told me to keep going”—Hugo	
For me, it’s really about making my dad proud—that’s part of what drives me—Adam	
“My dad’s my biggest fan—he hasn’t missed many games. It’s a huge support, but at the same time, it adds some pressure. You just don’t want to let him down”—Lucas	The hockey dad
“Do I regret him getting a bit crossed with me? No, because if he hadn’t, I probably wouldn’t be here today”—Lucas	
“I saw my dad’s gear and thought, ‘This is what I want to do,’ so I guess I’ve never really complained about it”—Anton	
“My dad knows you don’t have to be the best at 15 to make it later. As long as I’m having fun, I’ll keep going”—Gustav	

**Table 5:** Exemplary quotes for the theme The Struggle Between Dream and Reality.

Quotes (Meaning Units)	Category
“My love for hockey will probably never fade”—Joel	The NHL dream
“When you’re a kid, it’s all about making it to the NHL and being the best in the world”—Axel	
“I really thought I was going to the NHL”—Adam	
“I felt that life is too short not to follow your dreams”—Lucas	
“But pretty quickly, you start to realize, that dream isn’t all that realistic”—Axel	Reality check
“The career doesn’t last forever—I get that, trust me”—Lucas	
“I know a lot of friends who quit because it just wasn’t their day—or even their year”—Adam	
“A lot of people quit when others start getting better and they’re no longer the best. That’s always been the way I’ve pushed myself to work harder”—Gustav	
“Then you come to X, where everyone’s about the same level, and you’re not the star anymore”—Ville	
Do I just break down and keep going, or do I give up because things didn’t turn out the way I wanted?”—Lucas	Self-Doubt and Performance Anxiety
“Between my first and second year of high school, things hadn’t gone the way I expected, they’d gone really badly. It felt like everyone else was developing faster than me, and at that point, it just didn’t feel worth it anymore. I stopped caring”—Anton	
“There were many times when you didn’t want to get up in the morning”—Hugo	

## Data Availability

The data that support the findings of this study are included in the article. Original transcripts are not publicly available due to ethical restrictions.

## Supplementary Materials

The following supporting information can be downloaded at: https://www.mdpi.com/article/10.3390/sports14060235/s1, Supplementary File S1: The interview Guide.

## Abbreviations

The following abbreviations are used in this manuscript:

LTAD	Long-Term Athlete Development
DMSP	Developmental Model of Sport Participation
HA	HockeyAllsvenskan
SHL	Swedish Hockey League
NHL	National Hockey League
