# Supplementary material for: Formed on Ice: A Qualitative Study of Motivation, Pressure, and Identity in Early Ice Hockey Specialization
Source: Sports (Basel). 2026 Jun 8;14(6):235. doi: 10.3390/sports14060235 (PMC13306565; doi:10.3390/sports14060235)
Supplement: Supplementary file 1 [file sports-14-00235-s001.zip › sports-4254127-supplementary.pdf]

Supplementary file

The interview Guide

Background

- Who are you? (age, background in hockey up until today; club affiliation, levels, number of years as an active player)

- At what age did you start playing ice hockey?

- Have you practiced other sports alongside hockey? If yes, which sports and at what level?

- When did you stop participating in other sports to fully commit to hockey?

The Early Hockey Career

- How did you experience your everyday life as a young person in relation to the choice of early specialization?

- How did you experience the training volume and physical load while growing up?

- What factors contributed to your continued commitment to hockey?

Experiences and perceptions of early specialization

- What is your own perception of the concept of early specialization?

- Do you see any advantages or disadvantages with early specialization in hockey? (If yes, what are they and how do they affect the athlete?)

- What are your thoughts on the relationship between early specialization and reaching a higher level in hockey?

Social support and motivation

- Can you describe how, if at all, your involvement in hockey has influenced your social life or participation in social activities?

- In what ways, if any, has early specialization influenced your relationships with others?

- Do you feel that you’ve received support from your loved ones? (If yes, how? If no, how do you feel about that?)

- What motivated you to fully commit to hockey?

- Has your motivation changed over time? (If yes, in what way?)

- Can you describe any experiences you've had with changes in motivation, and how you responded to them?

- In what ways, if any, have people around you, such as coaches, leaders, family, or teammates, impacted your motivation?

Reflections and the Future

- If you had the option to change something in your sports career, would you? (If yes, what would you change?)

- What advice would you give to parents and young hockey players regarding the choice to specialize early?

- How should coaches, leaders, and clubs work with young players to best support their future careers (development)?

- Is there anything else you would like to add or highlight about this topic?
